# The Relevance of Monoclonal Antibodies in the Treatment of COVID-19

**DOI:** 10.3390/vaccines9060557

**Published:** 2021-05-26

**Authors:** Anabel Torrente-López, Jesús Hermosilla, Natalia Navas, Luis Cuadros-Rodríguez, José Cabeza, Antonio Salmerón-García

**Affiliations:** 1 Department of Analytical Chemistry, Science Faculty, Biohealth Research Institute (ibs.GRANADA), University of Granada, E-18071 Granada, Spain; anabeltl@ugr.es (A.T.-L.); herfer@correo.ugr.es (J.H.); lcuadros@ugr.es (L.C.-R.); 2 Department of Clinical Pharmacy, Biohealth Research Institute (ibs.GRANADA), San Cecilio University Hospital, E-18012 Granada, Spain; jose.cabeza.sspa@juntadeandalucia.es (J.C.); asalgar6@gmail.com (A.S.-G.)

**Keywords:** COVID-19 treatment, clinical trials, monoclonal antibodies, non-SARS-CoV-2 specific, SARS-CoV-2 specific

## Abstract

Major efforts have been made in the search for effective treatments since the outbreak of the COVID-19 infection in December 2019. Extensive research has been conducted on drugs that are already available and new treatments are also under development. Within this context, therapeutic monoclonal antibodies (mAbs) have been the subject of widespread investigation focusing on two target-based groups, i.e., non-SARS-CoV-2 specific mAbs, that target immune system responses, and SARS-CoV-2 specific mAbs, designed to neutralize the virus protein structure. Here we review the latest literature about the use of mAbs in order to describe the state of the art of the clinical trials and the benefits of using these biotherapeutics in the treatment of COVID-19. The clinical trials considered in the present review include both observational and randomized studies. We begin by presenting the studies conducted using non-SARS-CoV-2 specific mAbs for treating different immune disorders that were already on the market. Within this group of mAbs, we focus particularly on anti-IL-6/IL-6R. This is followed by a discussion of the studies on SARS-CoV-2 specific mAbs. Our findings indicate that SARS-CoV-2 specific mAbs are significantly more effective than non-specific ones.

## 1. Introduction

Coronavirus disease 2019 (COVID-19), caused by severe acute respiratory syndrome coronavirus 2 (SARS-CoV-2), has emerged as a new infectious disease which has reached pandemic proportions. This coronavirus belongs to the *Betacoronavirus* genus in the Coronaviridae family, together with other previously identified coronaviruses, such as SARS-CoV and MERS-CoV. These viruses have a positive-sense RNA genome which encodes structural and non-structural proteins [1]. SARS-CoV-2 transmission is mainly mediated by respiratory droplets and aerosols and most infected patients are asymptomatic or present mild symptoms, such as fever, cough, dyspnoea, diarrhoea, muscle pain, sore throat, headache, and loss of smell and/or taste. However, about 20% of patients undergo a serious illness with dyspnoea, pneumonia, and supplemental oxygen requirements. The most seriously ill patients can suffer respiratory failure and cardiopulmonary collapse or shock that can lead to death [2]. In response to this global emergency, a wide range of therapeutic agents such as chloroquine, hydroxychloroquine, antivirals, antibodies, corticosteroids, or convalescent plasma among others have been or are currently being evaluated for the treatment of COVID-19 [3], in addition to the development of vaccines. Unfortunately, not all these agents have proved successful and some, such as chloroquine, hydroxychloroquine and several antivirals, have already been discarded as possible treatments [4,5,6].

**Figure 1:**
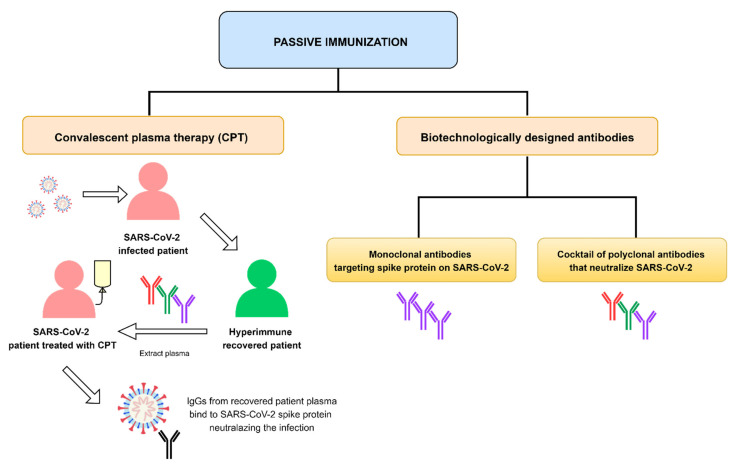
Different strategies to guarantee passive immunization using antibodies.

One of the strategies considered for defeating COVID-19 is passive immunotherapy (Figure 1). There are two ways to guarantee passive immunization: (i) via natural antibodies using convalescent plasma therapy (CPT) in which plasma is extracted from a hyperimmune patient and transfused into a COVID-19 patient; or (ii) via antibodies that are biotechnologically designed, i.e., therapeutic monoclonal antibodies (mAbs) or a cocktail of polyclonal antibodies (pAbs) [7]. Of these two passive immunization strategies, the use of mAbs offer the most innovative approach to the prevention and treatment of infectious diseases, such as COVID-19, where current research aims at developing treatments based on specific mAbs to block and/or neutralize SARS-CoV-2 in infected patients [8]. In addition, already available mAbs have been used off-label based on the knowledge acquired during the pandemic regarding the pathogenesis of the disease. Therefore, the characteristic of mAbs made them perfectly suitable for the treatment of COVID-19 [9].

The off-label use of drugs can be defined as their use for a non-officially approved condition. It also refers to the use of drugs with an unapproved dosage, route of administration, or in an unlicensed combination regimen [10]. Off-label administration of drugs to treat COVID-19 is an extensive practice. However, this is not the first time that mAbs have been prescribed off-label. Several mAbs have proven safe and effective for treatments not indicated in their respective Summary of Product Characteristics (SPC). One example is bevacizumab: an anti-cancer biotherapeutic which is currently widely administered intravitreally to treat age-related macular degeneration (AMD) instead of the approved drug, ranibizumab [11]. Although both biotherapeutics have similar efficacy and safety, bevacizumab is now preferred due to its better cost–benefit ratio.

**Figure 2:**
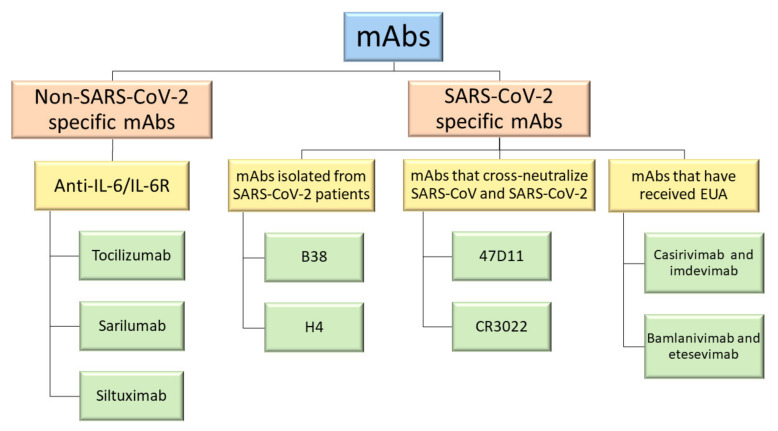
Relevant mAbs used to treat COVID-19 deeper discussed in this review.

Several clinical trials are currently being conducted to test the efficacy and safety of different mAbs for the treatment of COVID-19, some of which are already being administered in hospitals while others are under evaluation [12]. Many of them target immune system responses (non-SARS-CoV-2 specific mAbs) while others are designed to neutralize the SARS-CoV-2 protein structure (SARS-CoV-2 specific mAbs) (Figure 2) [7]. This paper aims to present the state of the art on the most investigated mAbs currently under consideration for the treatment of the novel coronavirus disease.

## 2. Non-SARS-CoV-2 Specific Monoclonal Antibodies

The mAbs that are currently being used in hospitals to treat COVID-19 focus on the immune responses provoked by the virus, which can affect the severity of COVID-19 disease [7]. One of these immune responses involves the sudden release of large numbers of certain cytokines into circulation, in what is known as a ‘cytokine storm’, a life-threatening systemic inflammatory syndrome [13]. IL-6 is one of the key pro-inflammatory cytokines found in COVID-19 patients, which is why anti-IL-6/IL-6R biological drugs have been used for the treatment of this disease from the beginning. The mAbs selected to treat COVID-19 patients with high IL-6 levels include tocilizumab, sarilumab, and siltuximab. Several clinical trials are currently underway to test their efficacy, which has yet to be fully proven [2].

Clinical trials are also ongoing with other non-SARS-CoV-2 specific mAbs whose therapeutic targets are not IL-6/IL-6R, such as bevacizumab, clazakizumab, eculizumab, emapalumab, gimsilumab, itolizumab, mavrilimumab, meplazumab, nivolumab, pembrolizumab, etc. [2,12,14]. The therapeutic, anti-IL-1R protein, anakinra, has also been used [15]. No conclusive results have been obtained with any of them as yet. Due to their widespread use, in this paper we will be focusing specifically on the anti-IL-6/IL-6R mAbs and their use in the treatment of the COVID-19-associated cytokine storm.

### 2.1. Tocilizumab (TCZ)

TCZ is a humanized IgG1 mAb that targets both soluble and membrane-bound IL-6 receptors and inhibits the signal mediated by them. It is frequently used in the treatment of different autoimmune diseases such as rheumatoid arthritis and systemic juvenile idiopathic arthritis [16].

Soon after the pandemic outbreak, off-label use of mAbs became a standard part of care in patients with COVID-19 in different parts of the world. Of the arsenal of therapeutics proposed as anti-COVID-19 candidates, the anti-IL-6 mAbs seemed to be useful in severe patients. Of these, TCZ has been the most frequently administered in hospitals due to its greater supply, its longer history on the market and its availability in intravenous (IV) pharmaceutical format [17]. Its efficacy and safety in the treatment of COVID-19 are beginning to be assessed in a large number of observational studies, and also in various randomized clinical trials. To date, a total of 78 clinical trials have been registered [18].

Systematic reviews and meta-analyses have already been published which bring together the results from the clinical studies found in the literature. Boregowda et al. [19] published a systematic review and meta-analysis of studies that evaluated the benefits of TCZ in reducing mortality in severe COVID-19 patients as compared to similar patients who only received the standard treatment. This review included a total of 16 articles, most of them retrospective. The authors found statistical differences between the mortality rate of the TCZ group (22.4%) and that of the control group (26.21%), leading them to conclude that TCZ administration might reduce the mortality in severe COVID-19 patients, while stressing the need to conduct larger randomized clinical trials (RCT). However, the results of this meta-analysis have not been certified by peer-review. Berardicurti et al. [20] published a systematic review and meta-analysis evaluating the effect of TCZ administration on mortality. They included 22 studies, most of them retrospective. They reported a reduction in the odds of mortality in TCZ-treated patients when compared to the control group. Cortegiani et al. [21] also published a systematic review of 31 clinical studies; most of them single-center and retrospective, none of them randomized. Although some of these studies claimed that TCZ might be effective and could be related to better outcomes, they emphasized the observational nature of the studies and concluded, therefore, that they had a degree of inaccuracy due to different bias sources, mainly due to confounding. Of the 31 studies they analyzed, 15 had a comparison control group without TCZ, and 5 of these 15 were classified as presenting a serious risk of bias while the rest were considered of moderate risk. Khan et al. [15] published a systematic review and meta-analysis of studies assessing the effects of four anti-IL-6 agents against COVID-19. They primarily evaluated severity on an Ordinal Scale measured at day 15 from intervention and in terms of the number of days to hospital discharge. They also evaluated overall mortality as a secondary endpoint. TCZ was analyzed in a total of 60 studies (12 prospective with control arm, 8 prospective without control arm, and 40 retrospective). In the prospective studies TCZ was related to a lower relative risk of mortality. However, its effects were inconclusive for other outcomes.

There have also been various reviews analysing the results of the RCTs conducted to evaluate the efficacy of TCZ in the treatment of COVID-19 (Table 1). A recently published review performed a meta-analysis of randomized clinical trials evaluating the effect of TCZ on all-cause mortality as the first outcome and a combined endpoint of requirement of mechanical ventilation as the second outcome [22]. In this research the authors included six clinical trials that were assessed for their risk of bias and were classified as of low and moderate risk. These RCTs included a total of 1177 patients who were randomly selected and administered TCZ and 880 patients who were randomized to the control group and did not receive TCZ. This meta-analysis demonstrated that the administration of TCZ reduced the likelihood of progression to mechanical ventilation and/or all-cause mortality among hospitalized patients with COVID-19. However, no clear benefits on mortality as an endpoint were reported with the administration of TCZ to hospitalized patients with COVID-19.

The National Institute for Health and Care Excellence (NICE) periodically updates a summary of the existing evidence on clinical trials evaluating the efficacy and safety of TCZ in the treatment of COVID-19 [23]. It also includes a section about the limitations of such studies. The evidence comes from five clinical studies conducted on hospitalized patients with COVID-19 pneumonia [24,25,26,27]. These studies were included in the meta-analysis discussed above [22]. They include evidence from another study by Veiga et al. [28], who performed a randomized open-label clinical trial (NCT04403685) in which they evaluated the use of a single dose of IV TCZ (8 mg/kg) plus standard care as compared to standard care alone. They concluded that TCZ plus standard care did not improve clinical outcomes at day 15 compared to standard care alone, and it might increase mortality in patients with severe or critical COVID-19. Gordon et al. [29] conducted a Randomized, Embedded, Multifactorial Adaptive Platform Trial for Community-Acquired Pneumonia (REMAP-CAP; NCT02735707) for evaluating the use of anti-IL-6 mAbs, i.e., TCZ and sarilumab in the treatment of critical cases of COVID-19. The main conclusion they reached was that the treatment with the IL-6 receptor antagonists (TCZ and sarilumab) improved outcomes, including survival. As a consequence, the National Health Service (NHS) encouraged doctors to consider prescribing either TCZ or sarilumab in the treatment of Intensive Care Unit (ICU) patients with COVID-19 pneumonia [17]. A short while ago, Horby et al. [30] published their preliminary results from a randomized, controlled, open-label clinical trial (RECOVERY; NCT04381936) performed in the UK. They concluded that TCZ administered intravenously in patients with hypoxia and systemic inflammation, improved survival and other secondary outcomes. Preliminary evidence from these two RCTs highlight a possible benefit in adults who have been hospitalized with severe COVID-19 and have clinical evidence of progressive disease (hypoxia and systemic inflammation) [30]; or with severe COVID-19 who are critically ill and receiving respiratory or cardiovascular organ support in an intensive care setting [29]. The most recent study was conducted in India [31]. The results of this open-label, multicenter, randomized, controlled, phase 3 trial showed that the primary and secondary endpoints were not significantly different between TCZ plus standard care and standard care alone. Consequently, they did not support routine use of TCZ in adults with COVID-19.

### 2.2. Sarilumab

Sarilumab is a human IgG1 mAb that targets both soluble and membrane-bound IL-6 receptors (IL-6Rα), and it inhibits IL-6-mediated signalling. It is indicated, in combination with methotrexate, for the treatment of moderate to severe rheumatoid arthritis in adult patients [32].

Although sarilumab is also an anti-IL-6 therapeutic agent, it has not been the subject of as much research as TCZ. To date a total of 17 clinical trials are ongoing [18,33]. Of these, five have been completed. Limited published studies exploring the benefits of this therapeutic against COVID-19 are available [15]. The first evidence came from observational studies. Khiali et al. [34] reviewed the potential use of sarilumab in the treatment of COVID-19 among other issues. They included four studies conducted in Italy: a clinical series study [35]; an observational clinical cohort study [36]; an open-label observational study [37]; and a retrospective case series [38]. Although the treatment with sarilumab 400 mg seemed to be safe, no clear evidence could be obtained from these studies and the authors therefore concluded that further clinical trials were necessary. The systematic review and meta-analysis done by Khan et al. [15] discussed five prospective studies that included a total of 389 participants who received sarilumab. This research concluded that there is a lack of evidence regarding the efficacy of sarilumab and highlighted the need for further studies.

Very recently, Castelnovo et al. [39] provided new evidence on the use of sarilumab to improve prognosis and reduce hospitalization times and mortality in COVID-19 pneumonia. Although their study was monocentric and retrospective, the authors argue that sarilumab seemed to be effective in the treatment of medium to severe forms of COVID-19 pneumonia, reducing the mortality risk driven by multi-organ failure, acting at the systemic level, and reducing inflammation levels and, therefore, microvascular complications.

RCTs are also being conducted to evaluate the efficacy of sarilumab in the treatment of COVID-19. In September 2020, Sanofi announced its phase III clinical trial results: sarilumab at a dose of 200 mg or 400 mg in severely or critically ill patients hospitalized with COVID-19 did not meet either its primary endpoint or its key secondary endpoint when compared to a placebo combined with standard hospital care [40]. The NICE also includes updated evidence on the benefits of sarilumab in the treatment of COVID-19. This site includes one prepublication [29] which suggests that sarilumab is beneficial in adults with severe COVID-19 [41]. The latest published literature on the use of sarilumab to treat COVID-19 includes a study conducted by Lescure et al. [42], who carried out a multinational, randomized, adaptive, phase 3, double-blind, placebo-controlled trial (NCT04327388), which involved SARS-CoV-2 positive patients with pneumonia who required oxygen supplementation or intensive care. Patients were randomized on a 2:2:1 ratio, receiving IV sarilumab 400 mg, sarilumab 200 mg, or placebo. This trial failed to demonstrate the efficacy of sarilumab in patients hospitalized with COVID-19 and receiving supplemental oxygen (Table 1).

### 2.3. Siltuximab

Siltuximab is a chimeric human-murine mAb that prevents human IL-6 from binding to both soluble and membrane IL-6 receptors (IL-6R). It is indicated for the treatment of multicentric Castleman’s disease (MCD) in adult patients [43].

Currently, there are three ongoing and one completed clinical trial that involve siltuximab [18]. Gritti et al. [44] conducted an observational, control cohort, single-center study (NCT04322188) on a total of 60 COVID-19 patients to assess their mortality rate. A total of 30 patients were allocated to the siltuximab treatment cohort group and the other 30 patients were placed in the control group. The mortality rate was assessed on day 30 of the study and was found to be significantly lower in siltuximab-treated patients than in the control group. This study therefore showed that the administration of siltuximab could be beneficial for COVID-19 patients with rapid progression to respiratory failure requiring ventilatory support, as it reduces the hyperinflammation caused by the cytokine storm, which is associated with severe disease. However, the authors recommended that their findings be validated in a randomized controlled clinical trial.

## 3. SARS-CoV-2 Specific Monoclonal Antibodies

**Figure 3:**
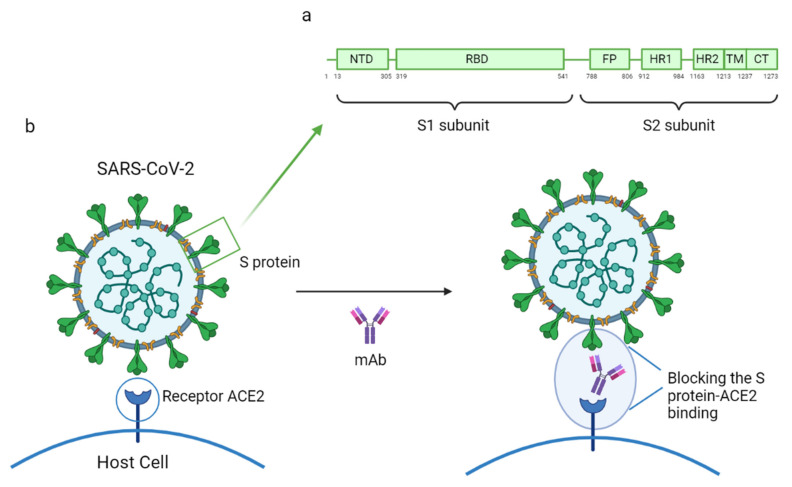
(**a**) Mechanism of action of a mAb by blocking the SARS-CoV-2 S protein and human ACE2 receptor binding; (**b**) structure of the SARS-CoV-2 S protein. This figure was composed using BioRender (available at: https://biorender.com/. Accessed on 12 May 2021).

Phylogenetic analysis has shown that SARS-CoV and SARS-CoV-2 are very similar in genetic and structural terms. This helped scientists understand the pathogenesis of COVID-19 and provided a basis for initial research. Indeed, the glycosylated spike (S) proteins from both coronaviruses have a primary amino acid sequence homology of 77.5% [7,8]. The S proteins are located on the surface of these coronaviruses and are a key component in infection. They mediate viral host cell entry by binding to the host cell receptor angiotensin-converting enzyme 2 (ACE2). The SARS-CoV-2 S protein is composed of 1273 amino acids (aa) and consists of three segments: an extracellular N-terminus, a transmembrane (TM), and a short intracellular C-terminal segment. A signal peptide is located at the N-terminus domain which consists of a few aa (1–13 residues). The rest of the protein is divided into two large regions: S1 and S2 subunits. The S1 subunit (14–685 aa) is responsible for receptor binding and consists of an N-terminal domain (NTD) and a receptor-binding domain (RBD). The S2 subunit (686–1273 aa) comprises the fusion peptide (FP), heptapeptide repeat sequence 1 (HR1), HR2, TM domain, and cytoplasm domain, and is involved in the fusion of the viral and host cell membranes and the consequent release of the viral genome into the host cell (Figure 3a). The RBD region is considered a critical target for neutralizing antibodies (nAbs) since it allows the spike protein to bind to the cell receptor ACE2. A deeper knowledge about the structure and configuration of the SARS-CoV-2 S protein can be found in references [45,46].

The mAbs specifically designed to combat SARS-CoV-2 can be classified into three groups based on their respective objectives: (1) inhibiting virus attachment and entry by targeting either the virus structure or host receptors; (2) interfering with virus replication and transcription; and (3) hindering various stages of the immune system response [7]. Most of the mAbs currently under development target the S protein, which the virus uses to enter host cells [47]. Some reports highlighting the efficacy of specific mAbs against COVID-19 have already been published. These reports propose B38, H4, 47D11, and CR3022 as potential SARS-CoV-2 specific mAbs as they have demonstrated significant capacity to prevent infection by the virus. All these mAbs act by binding to the receptor-binding domain (RBD), therefore inhibiting the union between the virus and the human-ACE2 receptor (Figure 3b). It was also noted that B38 and H4, which had been isolated from a convalescent patient, bind exclusively to SARS-CoV-2 RBD, while 47D11 and CR3022 were tested against SARS-CoV and have demonstrated their ability to cross-neutralize SARS-CoV and SARS-CoV-2 [7,8,48]. In Table 2 are shown the binding site and the mechanism of action of the SARS-CoV-2 specific mAbs discussed in this review.

### 3.1. mAbs Isolated from SARS-CoV-2 Patients

Plasma from convalescent COVID-19 patients is regarded as an important source of a variety of specific SARS-CoV-2 mAbs that directly target SARS-CoV-2 neutralization. However, the infusion of convalescent plasma in late stages of the illness has proved unsuccessful at improving patient condition. It was therefore decided to test the potential benefits of plasma administration in an earlier phase of the illness. To this end, a randomized, double-blind, placebo-controlled clinical trial was conducted to evaluate the administration of high-titer convalescent plasma within 72 h after the onset of mild COVID-19 symptoms. In this study, severe respiratory disease was considered the primary end point and a total of 160 patients were enrolled. Despite the fact that this trial had to be suspended, the results showed a reduction in COVID-19 progression in older adults who received early administration of convalescent plasma [49].

In order to find specific mAbs to combat this coronavirus, various antibodies have been isolated from the blood of convalescent patients. Some of the findings are discussed below.

Wu et al. [48] isolated four human-origin mAbs from a convalescent COVID-19 patient which were given the names B5, B38, H2, and H4. All of these mAbs were able to block the union between the S protein RBD domain and the ACE2 human cell receptor. These authors also found out that the newly discovered mAbs bound to SARS-CoV-2 RBD but not to SARS-CoV RBD, which suggests that the epitopes of both receptors are immunologically distinct. In order to test the ability of each mAb to perform their neutralizing activity, the authors carried out a competition assay using biolayer interferometry (BLI). Their results showed that B38 and H4 displayed complete competition with ACE2 for RBD binding. B5, however, showed only partial competition and H2 did not compete with ACE2. In order to identify the epitope targeted by B38 and H4, they performed an epitope competition assay. The results suggested that these mAbs recognize different epitopes on RBD. This study concluded that B38 and H4 are promising candidates for use as part of antibody-based prophylactic and therapeutic COVID-19 treatment.

For their part, Zhou et al. [50] isolated another antibody, EY6A, from a convalescent COVID-19 patient. ELISA assays showed that EY6A bound to SARS-CoV-2 S protein and cross-reacted SARS-CoV with lower affinity. Using surface plasmon resonance (SPR), they determined that this mAb bound to SARS-CoV-2 RBD in a site that was spatially separate from that of ACE2. They also conducted three different neutralization assays which demonstrated that SARS-CoV-2 was highly neutralized by EY6A, which means that this antibody is also a possible candidate for use in the treatment of COVID-19.

### 3.2. mAbs That Cross-Neutralize SARS-CoV and SARS-CoV-2

Other studies have shown that 47D11 and CR3022 can bind to a highly conserved epitope in the RBD of the S protein in both coronaviruses [51,52]. Preclinical studies are being carried out with these mAbs in order to demonstrate their ability to cross-neutralize SARS-CoV and SARS-CoV-2.

Wang et al. [53] developed an ELISA-cross-reactivity assay with the aim of identifying cross-neutralizing SARS-CoV and SARS-CoV-2 mAbs. Only one antibody (47D11) showed cross-neutralizing activity. Furthermore, the ELISA assays demonstrated that 47D11 targeted the RBD of both coronaviruses and their bindings displayed similar affinities. The authors explain this cross-neutralization as a consequence of 47D11 binding to a conserved epitope in the RBD. They concluded that this mAb has good potential in the prevention and treatment of SARS-CoV-2 infected patients in either mono- or combined therapy.

Tian et al. [54] reported for the first time that CR3022, a SARS-CoV-specific mAb which was previously isolated from the blood of a convalescent SARS-CoV patient, could potentially bind to SARS-CoV-2 RBD. This result was determined by ELISA and BLI assays which confirmed that CR3022 could be considered as a candidate therapeutic for the treatment and prevention of COVID-19. Nevertheless, this study revealed that some potent SARS-CoV-specific neutralizing antibodies are unable to bind to the SARS-CoV-2 S protein, a result that was attributed to the differences between the C-terminus residues of the two coronaviruses. These differences had a significant impact on the cross-neutralizing activity of SARS-CoV-specific antibodies, which highlights the need to develop novel SARS-CoV-2-specific mAbs. Yuan et al. [55] determined the complex crystal structure of CR3022-SARS-CoV-2 RBD. Their outcomes showed that this mAb targets a highly conserved epitope which is responsible for the cross-reactive binding to SARS-CoV and SARS-CoV-2. However, despite the fact that CR3022 is able to bind to both coronaviruses, it does not neutralize SARS-CoV-2. Wrobel et al. [56] supported these findings by using cryo-electron microscopy to study the mechanism by which CR3022 and SARS-CoV-2 S protein bind together. Their results confirmed that some rearrangements in the S1 domain are needed for such a union to occur, which result in dissociation of the S protein. They also reported that CR3022 did not neutralize SARS-CoV-2. Later, Wu et al. [57] performed a study involving CR3022 which again highlighted the different affinity of this mAb for the two types of coronavirus under consideration. Therefore, the authors studied the molecular basis leading to the difference in the binding affinity and neutralizing potency of CR3022 to SARS-CoV and SARS-CoV-2. They performed mutagenesis and binding experiments and demonstrated that the amino-acid located at residue 384 of the SARS-CoV-2 RBD structure was responsible for the different affinity. Moreover, given that the CR3022 epitopes in SARS-CoV and SARS-CoV-2 differ by four residues, the authors decided to use four SARS-CoV-2 mutants. This study showed that a single mutation (SARS-CoV-2 P384A mutant) improved the neutralization of SARS-CoV-2 with a similar potency to that shown in the neutralization of SARS-CoV. Their findings shed light on the cross-reactivity that some antibodies such as CR3022 can exhibit. In order to boost the therapeutic potential of CR3022, Atyeo et al. [58] dissected various strategies based on the use of selectively engineered Fc variants to enhance its functionality. However, their results showed an increased pathology in the animal models used, while highlighting the need for strategic Fc engineering for the treatment of COVID-19.

Given that each mAb has different binding sites, it has been suggested that a combination therapy of two or more mAbs that recognize neutralizing and non-neutralizing epitopes could well be the most effective way of using mAbs to treat COVID-19, as a single therapy might not be sufficient [51].

### 3.3. mAbs That Have Received Emergency Use Authorization (EUA)

Recently, the FDA issued an EUA for certain mAbs undergoing clinical trials that have shown significant efficacy in COVID-19 infected patients. An EUA is different from FDA approval and is based on all the available scientific evidence. The potential benefits of the drugs that have received an EUA outweigh the potential risks when used for the treatment of COVID-19 in the authorized population. These mAbs have been authorized for use in non-hospitalized adult and pediatric patients (12 years of age or older weighing at least 40 kg) with mild to moderate COVID-19 but at high risk of disease progression and/or hospitalization. The authorized mAbs may not be administered to hospitalized COVID-19 patients or to patients who require oxygen therapy due to COVID-19, as no studies have been conducted on hospitalized COVID-19 patients, a fact made clear on the information sheets for these mAbs. Some studies even indicate that hospitalized COVID-19 patients who require high flow oxygen or mechanical ventilation could undergo worse clinical outcomes if treated with these mAbs [59,60,61].

The first mAb under clinical trial to receive an EUA (November 09, 2020) from the FDA was bamlanivimab (LY-CoV555) [62], which was specifically designed to prevent the SARS-CoV-2 S protein from binding to and entering the host cells. The bamlanivimab EUA is supported by an ongoing, randomized, double-blind, placebo-controlled, single-dose phase 2 clinical trial (NCT04427501) conducted amongst 452 non-hospitalized patients that were diagnosed with mild or moderate COVID-19. These patients were divided into four groups according to the dose of bamlanivimab or placebo they received via intravenous infusion: (1) 101 patients were assigned to 700 mg of LY-CoV555 monotherapy; (2) 107 patients were assigned to 2800 mg of LY-CoV555 monotherapy; (3) 101 patients were assigned to 7000 mg of LY-CoV555 monotherapy; and (4) 143 patients were assigned to the placebo group. The quantitative virologic endpoints and clinical outcomes were evaluated and the results from the interim analysis (at day 11) showed that the second treatment (group 2) was the only one that appeared to accelerate the natural decline in viral load over time. Moreover, the patients who received LY-CoV555 showed slightly less severe symptoms over the period from day 2 to 6 than those in the placebo group. The percentage of COVID-19 patients who were hospitalized or had to visit an emergency department was lower (1.6%) in the LY-CoV555 patients than in the placebo group (6.3%) [63].

Subsequently, on November 21, 2020, the FDA announced an EUA for casirivimab (REGN10933) and imdevimab (REGN10987) used in a combined cocktail called REGN-COV2 [64]. Both mAbs bind to non-overlapping epitopes of the SARS-CoV-2 S protein RBD and potently neutralize the entry of the virus into the host cells. In a previous preclinical study [65], in vivo efficacy of REGN-COV2 was assessed in two animal models, in which rhesus macaques and golden hamsters were used to evaluate mild and severe disease respectively. In this study, reduction in viral load in the upper and lower airways, reduction in virus-induced pathology (in rhesus macaques) and loss of weight (in hamsters) were assessed in order to evaluate the efficacy of combined therapy with casirivimab and imdevimab. The study concluded that REGN-COV2 successfully reduced virus load in the upper and lower airways. Furthermore, this combined therapy decreased virus-induced pathological sequelae when administered in rhesus macaques and limited the weight loss in hamsters. Given the significant efficacy of REGN-COV2 shown in preclinical studies, a clinical trial (NCT04425629) was carried out in order to evaluate the decrease in viral load in symptomatic non-hospitalized COVID-19 patients and also to assess the safety and efficacy of this therapy. The main reason for employing a cocktail of mAbs was to reduce the risk of treatment-resistant mutant virus emergence. The first 275 patients included in this ongoing, multicenter, randomized, double-blind, phase 1–3 clinical trial were selected to describe the results of an initial analysis. All patients were randomly assigned: (1) 92 patients received 2.4 g of REGN-COV2; (2) 90 patients received 8.0 g of REGN-COV2; and 93 patients received a placebo. Results of the study revealed a reduction in the viral load when using this mAbs cocktail [66], on the basis of which the FDA decided to issue the aforementioned EUA.

The FDA later issued an EUA (February 09, 2021) for the combined administration of bamlanivimab (LY-CoV555) and etesevimab (LY-CoV016) [67]. In a similar way to bamlanivimab, etesevimab is specifically directed against the SARS-CoV-2 S protein and blocks the entry of the virus into the host cells. However, these mAbs bind to different, but overlapping, epitopes within the RBD of the SARS-CoV-2 S protein. This EUA relies on an ongoing, phase 2/3, randomized, double-blind, placebo-controlled clinical trial (NCT04427501) in which 577 non-hospitalized patients with mild to moderate COVID-19 symptoms were randomized to receive a single infusion of bamlanivimab, the combination treatment, or a placebo: (1) 101 patients were assigned to 700 mg of bamlanivimab; (2) 107 patients were assigned to 2800 mg of bamlanivimab; (3) 101 patients were assigned to 7000 mg of bamlanivimab; (4) 112 patients were assigned to 2800 mg of bamlanivimab and 2800 mg of etesevimab; and (5) 156 patients were assigned to placebo. The aim of this clinical trial was to determine the effect of the monotherapy and the combination therapy on viral load in mild to moderate COVID-19. The results drawn from this study conclude that the viral load reduction was statistically significant at day 11 in the combination therapy, compared with the placebo group. Nevertheless, bamlanivimab monotherapy showed no significant improvements in terms of viral load reduction [68].

On April 16, 2021, the FDA revoked the EUA for bamlanivimab monotherapy due to the fact that there had been an increase across the U.S. in the number of SARS-CoV-2 variants which are resistant to this treatment. The FDA therefore concluded that the known and potential benefits of bamlanivimab as a monotherapy no longer outweighed its known and potential risks. Importantly, alternative monoclonal antibody therapies remain available under EUA, including REGEN-COV, and bamlanivimab and etesevimab, administered together, for the same uses as had been previously authorized for bamlanivimab alone. Both therapies remain appropriate for treating patients with COVID-19 when used in accordance with the authorized labeling [69]. 

As regards the use of mAbs authorized by the FDA for emergency use in COVID-19 treatment and the recently initiated vaccination process, the Advisory Committee on Immunization Practices (ACIP) has recommended a precautionary measure as to date there are no data on the effectiveness and safety of either the Pfizer-BioNTech or Moderna COVID-19 vaccines (both mRNA vaccines) in SARS-CoV-2 infected patients who had previously received one of the aforementioned mAbs therapies. The ACIP suggests postponing the vaccination for 90 days after using mAbs for COVID-19 treatment. This recommendation is based on available data considered by the agency which suggests that there is a low risk of SARS-CoV-2 reinfection in the 90 days after initial infection and considering the estimated half-life of mAbs. The purpose of this measure is to avoid interferences between the mAbs used in the COVID-19 treatment and the vaccine-induced immune response [70,71,72].

### 3.4. How SARS-CoV-2 Mutations Could Affect the Efficacy of the Treatment with mAbs

Although specific mAbs are more promising in the treatment of COVID-19 than non-specific ones, some concerns are arising given the advent of new SARS-CoV-2 variants, such as B.1.351 and B.1.1.7. Recent publications [73,74] have discussed how new SARS-CoV-2 variants could affect the efficacy of the treatments with antibodies. It might seem logical that the efficacy of CPT treatment would be less impaired by the new variants due to the presence of antibodies with specificity for different S protein epitopes. However, it has been demonstrated in preclinical studies that S protein mutations escape from polyclonal serum [75] and CPT has reduced neutralizing activity against some viral variants [74]. Nevertheless, a preprint article dated last February has suggested that the variants identified in the United Kingdom and South Africa are more resistant to CPT [76].

With regard to mAbs, a combination of them could be necessary if the new variants are the results of high mutation rate in the virus. In the case of low mutation rates or highly conserved epitopes, a combined mAbs therapy could be not required. On the other hand, RNA viruses display high mutation rates due to the errors introduced by the RNA-dependent RNA polymerases (RdRp). However, coronaviruses encode a 3′ to 5′ exoribonuclease that helps to correct errors made by the RdRp during replication. This leads to a lower probability of escaping from antibody neutralization in comparison with other RNA viruses that do not encode this enzyme [75]. Focusing on mAbs directed to SARS-CoV-2, it has been very recently reported—April 2021—drug resistance in new variants, for example, to bamlanivimab as indicated above [69]. To date, a proper knowledge of the effect of new variants on the neutralizing capacity of mAbs is unknown [73]. 

## 4. Expert Opinion

In recent years, mAbs and Fc-fusion proteins have made it possible to take qualitative and quantitative steps forward in terms of therapeutic options for diseases with a clearly defined biological target, such as infectious diseases and, in this specific case, COVID-19. MAbs open up a new world in the field of passive immunization, which enables us to administer specific neutralizing antibodies against certain highly conserved epitopes of SARS-CoV-2 and also to obtain new drugs in record time, so that in just one year there are now more than 10 monoclonal antibodies in different stages of clinical development for use against COVID. Some of them are very promising and are almost ready for commercial launch.

## 5. Conclusions

Although mAb production is time-consuming and expensive, especially for use against new pathogens, they have been regarded as a good option for the treatment of COVID-19 since the beginning of the pandemic and many clinical trials on both non- and SARS-CoV-2 specific mAbs are currently ongoing. However, the outcomes of clinical trials for non-SARS-CoV-2 specific mAbs are proving controversial, as their efficacy has yet to be definitively demonstrated, whilst SARS-CoV-2 specific mAbs have demonstrated significant levels of efficacy. So far, two specific mAb-based COVID-19 treatments have received an EUA from the FDA. The first involves combined administration of casirivimab and imdevimab, and the second, combined administration of bamlanivimab and etesevimab. These clinical trials have therefore shown the importance of developing mAbs that are specifically targeted at SARS-CoV-2 rather than those aimed at the COVID-19-associated cytokine storm.

## Figures and Tables

**Table 1:** Published randomized clinical trials evaluating the efficacy of anti-IL-6 mAbs.

Drug	Authors	Design	Patients Enrolled	Regimen	Primary Outcomes	Main Findings
**TCZ**	Stone et al.	Prospective, multicenter, randomized, double blind, placebo-controlled trial	243 (162 TCZ group; 81 placebo group)	Standard care plus a single dose of either TCZ (8 mg/kg, IV, max 800 mg) or placebo	Intubation or death, assessed in a time-to-event analysis	TCZ was not effective in preventing intubation or death in moderately ill hospitalized patients with COVID-19
	Rosas et al.	Randomized, double-blind, placebo-controlled, multicenter study	452 (294 TCZ group; 144 placebo group)	A single IV infusion of TCZ (8 mg/kg, max 800 mg) or placebo plus standard care. A second infusion of TCZ or placebo could be administered 8 to 24 h after the first dose	Clinical status at day 28 on an ordinal scale ranging from 1 (discharged or ready for discharge) to 7 (death)	Administration of TCZ did not result in significantly better clinical status or lower mortality than placebo at day 28
	Salvarani et al.	Open-label randomized multicenter study	126 (60 TCZ group; 66 control group)	IV TCZ (8 mg/kg infusion, Max 800 mg) within 8 h from randomization, followed by a second dose after 12 h	Admission to the intensive care unit with invasive mechanical ventilation, death from all causes, or clinical aggravation documented by the finding of a PaO2/FIO2 ratio of less than 150 mmHg	No benefits in terms of disease progression were observed compared with standard care
	Hermine et al.	Cohort-embedded, investigator-initiated, multicenter, open-label, bayesian randomized clinical trial	131 (64 TCZ group; 67 usual care group)	IV TCZ (8 mg/kg infusion, max 800 mg) on day 1, with additional fixed dose of 400 mg as intravenous infusion on day 3 if required	Scores higher than 5 on the World Health Organization 10-point Clinical Progression Scale (WHO-CPS) on day 4 and survival without need for ventilation (including non-invasive ventilation) at day 14	TCZ did not reduce WHO-CPS scores to less than 5 at day 4 but might have reduced the risk of NIV, MV, or death by day 14. No difference was found in mortality on day 28
	Salama et al.	Randomized, double-blind, placebo-controlled, multicenter clinical trial	389 (249 TCZ group; 128 placebo group)	IV TCZ (8 mg/kg infusion, max 800 mg), with a second dose 8–24 h later if required)	Mechanical ventilation (invasive mechanical ventilation or extracorporeal membrane oxygenation) or death at day 28	TCZ reduced the likelihood of progression to the composite outcome of mechanical ventilation or death, but it did not improve survival
	Gordon et al.	Open-label, randomized, multifactorial, adaptive platform trial	747 (350 TCZ group; 397 control group)	TCZ (8 mg/kg, max 800 mg), was administered as an IV infusion over one hour; this dose could be repeated 12–24 h later at the discretion of the treating clinician	The primary outcome was an ordinal scale combining in-hospital mortality (assigned−1) and days free of organ support to day 21	In critically ill patients with COVID-19 receiving organ support in intensive care, treatment with TCZ improved outcome, including survival
	Veiga et al.	Multicenter, randomized, open label, parallel group, superiority trial	129 (65 TCZ group; 64 standard care group)	TCZ was administered as a single IV infusion at a dose of 8 mg/kg (max 800 mg)	The primary outcome, clinical status measured at 15 days using a seven- level ordinal scale, was analyzed as a composite of death or mechanical ventilation because the assumption of odds proportionality was not met	In patients with severe or critical COVID-19, TCZ plus standard care did not achieve better results than standard care alone in clinical outcomes at 15 days, and it might increase mortality
	Soin et al.	Open-label, multicenter, randomized, controlled, phase 3 trial	180 (90 TCZ group; 90 standard care group)	A single IV infusion at 6 mg/kg up to a maximum dose of 480 mg. An additional dose of 6 mg/kg (max 480 mg/kg) could be administered if required	The primary efficacy endpoint was the proportion of patients with progression of COVID-19 from moderate to severe or from severe to death up to day 14	Routine use of TCZ in patients admitted to hospital with moderate to severe COVID-19 is not supported. However, post-hoc evidence from this study suggests TCZ might still be effective in patients with severe COVID-19 and so should be investigated further in future studies
**Sarilumab**	Gordon et al.	Open-label, randomized, multifactorial, adaptive platform trial	442 (45 sarilumab group; 397 control group)	Sarilumab (400 mg) was administered once only as an IV infusion	The primary outcome was an ordinal scale combining in-hospital mortality (assigned−1) and days free of organ support to day 21	In critically ill patients with COVID-19 receiving organ support in intensive care, treatment with sarilumab, improved outcome, including survival
	Lescure et al.	Multinational, randomized, adaptive, phase 3, double-blind, placebo-controlled trial	416 (159 sarilumab 200 mg; 173 sarilumab 400 mg; 84 placebo)	Sarilumab 200 mg, 400 mg or placebo were administered as an IV infusion. A second dose could be administered within 24–48 h of the first dose if required	The primary endpoint was time to ≥2-point clinical improvement (7-point scale; range: 1 (death) to 7 (not hospitalized))	The efficacy of sarilumab was not demonstrated in patients hospitalized with COVID-19 and receiving supplemental oxygen

**Table 2:** Binding site and mechanism of action of SARS-CoV-2 specific mAbs discussed in this review.

Groups of Specific mAbs	Name	Binding Site and Mechanism of Action
MAbs isolated from SARS-CoV-2 patients	B5	SARS-CoV-2 RBD; partial competition with ACE2
	B38	SARS-CoV-2 RBD; complete competition with ACE2
	H2	SARS-CoV-2 RBD; no competition with ACE2
	H4	SARS-CoV-2 RBD; complete competition with ACE2
	EY6A	SARS-CoV-2 RBD and SARS-CoV RBD with lower affinity; site spatially separate from that of ACE2
MAbs that cross-neutralize SARS-CoV and SARS-CoV-2	47D11	SARS-CoV-2 and SARS-CoV RBD; conserved epitope in the RBD
	CR3022	SARS-CoV RBD and SARS-CoV-2 RBD with lower affinity; conserved epitope in the RBD. Do not neutralize SARS-CoV-2
MAbs that have received Emergency Use Authorization (EUA)	Bamlanivimab (LY-CoV555)	SARS-CoV-2 RBD; EUA revoked
	Casirivimab (REGN10933) and imdevimab (REGN10987) in a combined therapy	Non-overlapping epitopes of the SARS-CoV-2 RBD
	Bamlanivimab (LY-CoV555) and etesevimab (LY-CoV016) in a combined therapy	Different, but overlapping, epitopes of the SARS-CoV-2 RBD

## Data Availability

Not applicable.

## Abbreviations

ACE2: Angiotensin-Converting Enzyme 2; CPT: Convalescent Plasma Therapy; EUA: Emergency Use Authorization; FDA: Food and Drug Administration; ICU: Intensive Care Unit; IL-1R: Interleukin-1 Receptor; IL-6: Interleukin-6; IL-6R: Interleukin-6 Receptor; IV: intravenous; mAb: monoclonal antibody; pAb: polyclonal antibody; RBD: Receptor-Binding Domain; RCT: Randomized Clinical Trial; SARS-CoV: Severe Acute Respiratory Syndrome-Coronavirus; TCZ: tocilizumab.
